# Methods for visual mining of genomic and proteomic data atlases

**DOI:** 10.1186/1471-2105-13-58

**Published:** 2012-04-23

**Authors:** John Boyle, Richard Kreisberg, Ryan Bressler, Sarah Killcoyne

**Affiliations:** 1 Institute for Systems Biology, 401 Terry Ave N, Seattle, WA 98092, USA

## Abstract

**Background:**

As the volume, complexity and diversity of the information that scientists work with on a daily basis continues to rise, so too does the requirement for new analytic software. The analytic software must solve the dichotomy that exists between the need to allow for a high level of scientific reasoning, and the requirement to have an intuitive and easy to use tool which does not require specialist, and often arduous, training to use. Information visualization provides a solution to this problem, as it allows for direct manipulation and interaction with diverse and complex data. The challenge addressing bioinformatics researches is how to apply this knowledge to data sets that are continually growing in a field that is rapidly changing.

**Results:**

This paper discusses an approach to the development of visual mining tools capable of supporting the mining of massive data collections used in systems biology research, and also discusses lessons that have been learned providing tools for both local researchers and the wider community. Example tools were developed which are designed to enable the exploration and analyses of both proteomics and genomics based atlases. These atlases represent large repositories of raw and processed experiment data generated to support the identification of biomarkers through mass spectrometry (the PeptideAtlas) and the genomic characterization of cancer (The Cancer Genome Atlas). Specifically the tools are designed to allow for: the visual mining of thousands of mass spectrometry experiments, to assist in designing informed targeted protein assays; and the interactive analysis of hundreds of genomes, to explore the variations across different cancer genomes and cancer types.

**Conclusions:**

The mining of massive repositories of biological data requires the development of new tools and techniques. Visual exploration of the large-scale atlas data sets allows researchers to mine data to find new meaning and make sense at scales from single samples to entire populations. Providing linked task specific views that allow a user to start from points of interest (from diseases to single genes) enables targeted exploration of thousands of spectra and genomes. As the composition of the atlases changes, and our understanding of the biology increase, new tasks will continually arise. It is therefore important to provide the means to make the data available in a suitable manner in as short a time as possible. We have done this through the use of common visualization workflows, into which we rapidly deploy visual tools. These visualizations follow common metaphors where possible to assist users in understanding the displayed data. Rapid development of tools and task specific views allows researchers to mine large-scale data almost as quickly as it is produced. Ultimately these visual tools enable new inferences, new analyses and further refinement of the large scale data being provided in atlases such as PeptideAtlas and The Cancer Genome Atlas.

## Background

Systems biology is a field that relies on both technical and scientific innovations. The technical innovations enable new scientific questions to be asked, and these in return make further demands for advances in technology. High throughput experimentation has been the main driving force behind these advances, and has primarily encompassed measurement types.

Two measurement types that have seen a vast increase in utility and volume are high-throughput sequencing (HTS) and mass spectrometry based proteomics. The dramatic increase in the volumes of data are due to changes in instrumentation. In proteomics the adoption of new techniques, principally targeted approaches and high resolution instruments, means that there is a need to capture and mine vast quantities of high resolution spectra to enable the design of new assays. In genomics the cost of HTS is now at a scale where populations of genomes and transcriptomes can be captured, and this is being done in a number of projects (e.g. The Cancer Genome Atlas, 1000 Genome, International Cancer Genome Consortium). This paper outlines an approach to the development of visual tools that have been developed to allow for the direct mining and usage of data derived from these two technologies. Development of such tools requires an understanding of both the scale of data and the typical needs of the user in any exploration. The basic approach involves providing interactive high level overviews of the data, and then allowing for the selection and drill down into smaller data sets. Separate visualization tools are used at each level of data exploration and linked to enable users to quickly move between data views. The fast moving pace of research means that these tools must be put in place quickly, and so they have been built on top of a series of rapid application development technologies, and are delivered as web applications.

The example tools support two major systems biology projects, the PeptideAtlas [1] and The Cancer Genome Atlas (TCGA) [2]. The PeptideAtlas, encompasses SRM (Selected Reaction Monitoring) data across multiple species as well as shotgun based identifications, and the TCGA is a multi-institution effort to genomically characterize ten thousand cancer genomes across 20 different cancers.

## Methods

High throughput visualization tools are required to allow for the exploration of large data sets. The data sets in question consist of thousands of genomic sequences and protein mass spectra. As these atlases are relatively new, work to provide visual mining is in its infancy, however there has been a large amount of work in visualizations in the areas of network and gene expression visualization that is being adapted and learned from.

Systems biology generally requires the integrated analysis of different data types [3,4]. In systems biology the majority of information visualization has tended to focus on direct representations of networks [5]. This is due to the fact that networks are often used to describe the dynamics of living systems (as an integrated and interacting network of genes, proteins and biochemical reactions). Network visualization has been studied in a large number of disciplines (e.g. software visualization, including complex dynamics of systems [6,7] and the interactions of components [8,9]). The interest in networks and molecular interactions has resulted in the progression of network visualization techniques, in particular involving the portrayal of the complexities of relationship characteristics using number of edge techniques [10] (e.g. edge bundles [11] and edge lenses [12]). Additionally, the context in which parts of the network exist, either through the discovery of motifs or through semantic similarities, have been used to reduce the graph's visual complexity [13] (e.g. different levels of focus on a network [14], use of magic/document lens [15], and provision of identified landmarks to aid navigation [16,17]). These ideas are being applied to visualizations of systems biology networks [18,19].

Alternative metaphors for the representation of complex data have also been explored. Gene expression array based experiments have provided a rich area for the development of visual tools. In particular visualization of gene expression data has extended a number of popular n-dimensional data techniques: projecting high dimensional data down to two dimensions e.g. pair-wise scatter plots [20] and parallel coordinates [21]; encoding aspects of the data onto intrinsic and (non-positional) extrinsic properties (e.g. Spotfire [22]); and using dimension reduction techniques, which transforms the data onto a small number of dimensions (e.g. PCA, best-fit approaches [23-25]). Innovations have also arisen from these investigations in terms of improved representation of the data [26] and the provision of specialised visualisations which present the data in a relevant context (e.g. [27]). A number of visualization suites have been developed which combine these approaches (e.g. [28-30]).

Due to their scale and complexity the visualizations of large repositories of genomic and proteomic data do represent new challenges, however it is possible to use many of the general information visualization techniques. We provide details of the visualizations that have been used across data from both the PeptideAtlas and TCGA to enable users to explore the large-scale, highly dimensional data.

### Thousands of genomes

The Cancer Genome Atlas (TCGA) will, over the next three years, generate 10,000 patient genomic sequences across 20 different cancers. The goal is to provide a map of large-scale, genomic mutations, both between difference cancers (e.g. Ovarian and Glioblastoma) and across patients within a single cancer. Using these data, maps of normal variation, disease related disruptions and disease progression can be created for further analysis. Ultimately this atlas will provide a rich set of data to enable better characterization of disease sub types and the development of targeted therapies.

The data gathered by TCGA includes both full and exon-only genome sequences, epigenetic and transcriptomic data, and clinical information (e.g. age, clinical sub type). At the scale of thousands of patients this means that providing effective ways to visually explore this data is necessary for the development of useful analyses or in targeting areas for investigation. Such exploration requires the use of visual tools specifically adapted to data exploration at multiple scales. As the aim of TCGA is to provide genomic data across entire populations of patients and diseases, visual tools must enable exploration using specific knowledge (e.g. starting from a gene of interest) as well as providing for discovery of new information.

Various analyses are being performed across this data including pathway analysis, identification of functionally significant mutations and SNVs, tissue biopsy and imaging, microRNA regulation, and gene dosage analysis (to name a few). Each of these focuses on a different set of data within the atlas. Providing a generic visualization would result in a visual tool too abstract to be easily useful. Instead a set of tools targeted toward the specific analysis and underlying data is necessary. In developing tools for the analysis of gene disruptions, specifically structural variation, a linked set of interactive visual mining tools is used to directly compare the underlying genomic data. Each tool in the set is interactive so that genomic events can be discovered through exploration and used to find further information at each level (e.g. across cancers, across patients within a cancer, within a single patient).

**Figure 1:**
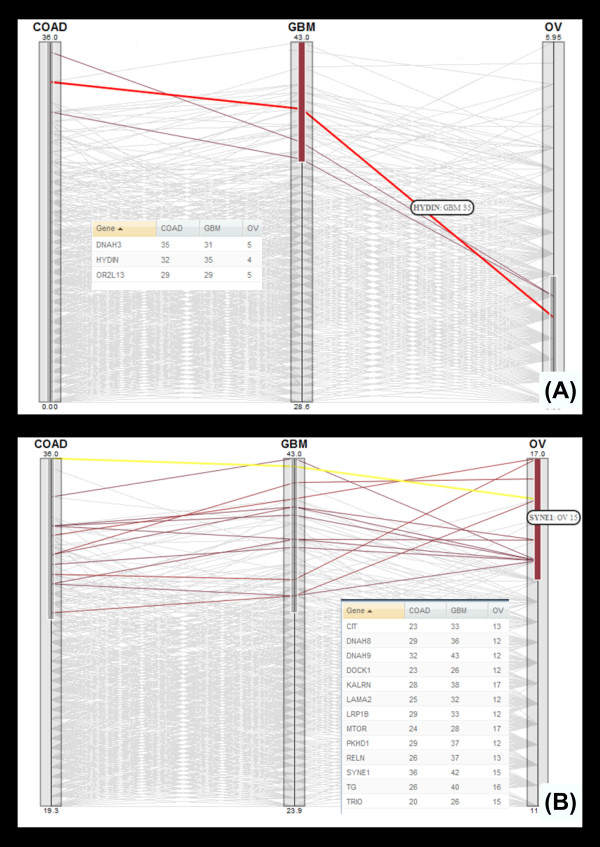
**Cancer comparator**. The cancer comparison macro view uses a parallel coordinates [31] to provide a cross disease comparison. In this case the visualizations are used to show the differences in gene disruptions, measured by examining structural aberration, between a carcinoma (Colon Cancer), a sarcoma (Ovarian Cancer) and GlioBlastoma (GBM). The values shown on each axis are the number of patients in which the specific gene has been disrupted. The visualization uses the Protoviz libraries, and provides blending and color coding to portray the trends of gene disruptions across the cancers. The visualization allows for range selection across the different axis, so that specific patterns across the cancers can be identified. The parallel coordinates allows for the queries to be performed directly on the data set. In the example (1a) the question being asked is which set of genes show a high level of structural aberration in GBM and a low level of structural aberration in ovarian cancer. The range selection tool has been used to select all genes that have shown aberrations in more than 27 (out of 43) patients in GBM, and also only show aberrations in less than 6 of the ovarian patients. The genes that show these characteristics are HYDIN, DNAH3 and OR2L13. HYDIN aberrations [34,35] are known to cause Hydrocephalus (water on the brain), and so the disruption of this gene in the brain produces a aberration that induces a survival physiological change. DNAH3 produces a Dynein protein and has been shown to be over expressed in ovarian cancer, under expressed in GBM [36] and also to be important in APC mutation based carcinogenesis in colon adenocarcinoma [37]. The OR2L13 olfactory gene is one without obvious function, however it is one of the main 44 recurrently mutated genes in this disease [38]. Figure 1b shows a second query, where the selection tool is used to identify all genes that show a high level of structural aberration across all three cancers. All the genes have been identified by others as being important in cancer and generally appear on multiple gene lists as complied by the MSKCC TCGA gene ranker tool [39]. The three genes that score lowest on this tool are PKHD1 [40] which is known to be involved in colorectal adenocarcinoma, DYNA9 which is involved in cilia transduction signals related to tumorgenesis important in Hedgehog and Wnt pathways [41], and SYNE1 which has recently been implicated in GBM [42]. SYNE1 is followed through the linked tools in Figures 2 and 3 to show the types of information that can be discovered and visualized.

**Figure 2:**
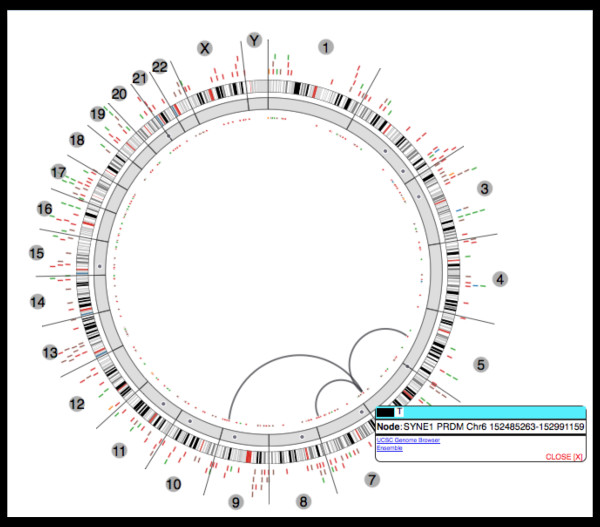
**Single cancer view**. The genome visualization provides a high level (macro) view to show similarities and differences across samples within a single cancer (Ovarian Cancer). This visualization is based around Circos [33], and allows for the display of high level aggregated features as concentric circles, with connecting arches showing identified associations, in this case common translocations. The concentric rings show, in this instance, information about the genes and karyotypes, and can include experiment information (e.g. identified mutations, methylation, expression). The associations are calculated, and in this instance show genes that have similar levels of disruptions. Selection of information allows for drill down into the related data sets, and filters can be applied which allow for control over the amount of information displayed.

**Figure 3:**
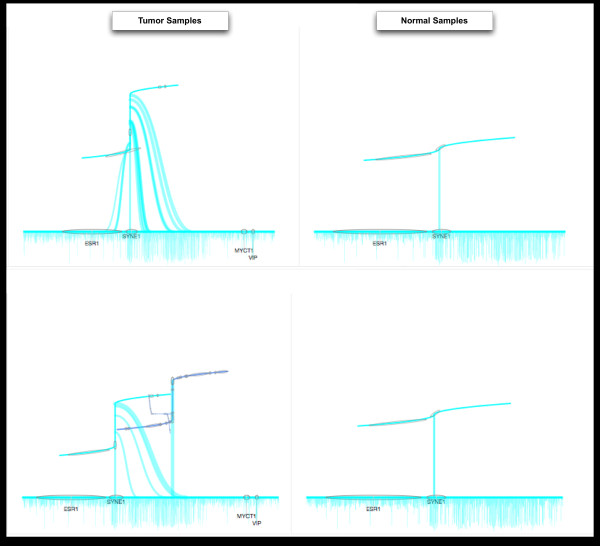
**Individual sample comparator**. The individual sample comparison tool allows for sets of patients to be explored. It shows disruptions at the gene or sub chromosome level and shows the complexity of gene disruptions between patients and normal/disease pairs. Using the cancer comparison (Figure 1) and genome focused (Figure 2) views, regions or genes of interest can be mined from hundreds of samples and then smaller sets of samples can be visually compared. In this instance on the right hand side are cancer samples, and on the left hand side are the matched normal tissues. The visualization displays the level of rearrangement at the chosen loci. The rearrangements can be complex and involve multiple crossovers or translations across different loci. To accommodate such complexity a nested layout procedure is used, where the main x-axis shows the scaffold chromosome, and the graph that is drawn directly from this shows represents how the rearrangement has resulted in connections between new non-contiguous portions of the chromosome (the thickness of the connecting curves gives an indication as to the portion of reads that show this level of structural variation). For complex multi-site rearrangements this branching procedure is repeated using nested graphs. The amount of disruption, and degree of gene fusion or similar, can then be visually compared. Color coding is used to show different chromosomes, and coverage information is displayed below the x-axis. The system is interactive so selecting on different loci will allow for further exploration filters can also be applied to change the patients being viewed.

The user starts from a high level parallel coordinates view [31] for exploration of differences in specific gene disruptions across multiple cancers (see Figure 1). Providing this view first requires that the underlying genomic data is processed with this exploration in mind. Structural variations (SV) were detected using a number of analysis tools such as Breakdancer [32], and the resulting data was then processed to remove biases. Coverage biases were removed using a biclustering technique, and mutual information was used to filter sets of genes that had strong dependencies (e.g. gene clusters such as protocadherins). The transformed data was then visualized using an interactive parallel coordinates view to show the proportion of patients where a given gene showed structural variation in each cancer. This cross-cancer view can then be mined for genes that are interesting across one or more cancers. The list of genes found to be interesting in this high-level visualization can be used to drill further into the data. The genes found in the high level view are then used to provide a visualization that focuses on a single cancer. The Circos [33] based tool shown in Figure 2 provides an interactive view of the similarities and differences across patient samples within a single cancer. The circular view enables an intuitive layout of chromosomes, while the connections between them display associations that can include various features, scores or other gene related information. This allows for the mining and integration of gene data with patient information to locate gene disruption locations in a manner that is easily understood. The final linked tool (Figure 3) provides a single sample level view to allow exploration of genes identified in the cross-cancer (Figure 1) and single cancer (Figure 2) tools. This zoom level focuses on a short chromosomal location (e.g. a gene) for a set of patients. The normal and cancer samples are shown side by side, allowing for the direct comparison of the structural variations within that location. At each level within the hierarchy of visual tools, the mining and filtering of the provided information enables a user to drill into the genes and samples that may provide the most information. Additionally, it should be noted that a different type of visualization was used at each level. This ensured that the scale of the data being viewed could be quickly comprehended (e.g. thousands of patients across multiple cancers vs. single patient sample pairs), enabling context appropriate views at each step of analysis.

### Millions of spectra

The PeptideAtlas repository contains thousands of experiments and is designed to provide a compendium of the likelihood of a given peptide being detected on mass spectrometers. The repository contains information from thousands of mass spectrometry experiments (millions of spectra) across numerous species, tissues and disease conditions. The goals in providing this repository are both to annotate genomic information with observable peptides, and to provide an integrated view on a given proteome so it can be used as the basis of Selected Reaction Monitoring (SRM) experiments. PeptideAtlas can be used to identify representative (proteotypic) peptides that are unique to an individual protein. Mining the data it contains makes it possible to identify which transition patterns could be used to uniquely identify any set of proteins in the proteome. This allows for the design of targeted proteomic experiments, where the experimenter defines a priori which proteins they wish to detect and using the atlas, find which specific transitions should be scanned for. Targeted approaches can monitor at low mass/charge (m/z) levels, and have been shown to detect protein concentrations at low copy number [43]. As SRM can be used on complex tissues, a minimum of separation chemistry is needed. This means that experiments can more accurately detect smaller amounts of protein in complex samples.

Mining this data requires the use of a number of integration strategies and information theoretic approaches to connect the peptide data with information from genomic sources (e.g. TCGA), disease literature (e.g. MEDLINE), and pathways (e.g. IntAct [44], MINT [45]). Providing visual tools that access this integrated data allows for refinement of biomarker or transition target lists from many thousands to the tens or hundreds that are detectable. The purpose is typically to identify peptides that will be suitable biomarkers for a specific disease or disease sub type. The mining tasks are rarely initiated without prior knowledge, instead they are typically initiated either through associations with other measurement types or through the literature information.

**Figure 4:**
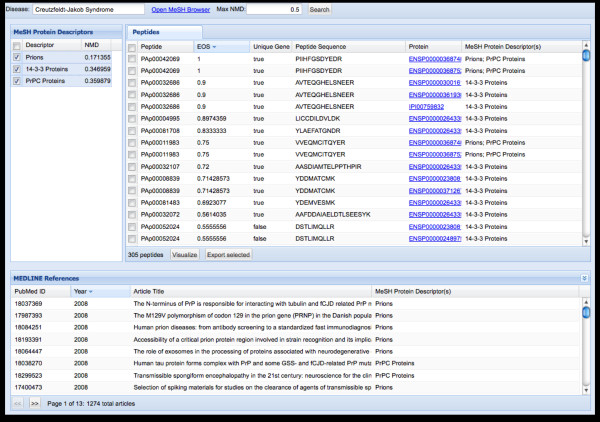
**mspecLINE Tool**. The mspecLINE tool [46] enables the mining of associations between literature information regarding specific diseases and observed peptide spectra. The resulting peptide lists can then be used to generate transition lists for new experiments. The user starts exploration from a specific disease and then all proteins associated with that disease are then discovered. Associations are discovered using an information theory based measure called Normalized Medline Distance. The evidence for the associations, and identified proteotypic peptides can then be retrieved or displayed in Cytoscape.

As with the genomic visualizations, those developed for exploring the PeptideAtlas are linked and interactive to allow for greater detail to be provided at the appropriately zoomed level. mspecLINE [46] (see Figure 4) is the first level mining tool which combines knowledge about human disease from MEDLINE with empirical data about the detectable human proteome from spectral libraries. It associates diseases with proteins by calculating the semantic distance, based on the co-occurrence of disease and protein terms in the MEDLINE bibliographic database, between annotated terms from a controlled biomedical vocabulary. This association allows for the exploration of relationships between human diseases and parts of the proteome that are detectable using current instrumentation. Given a disease, the tool will find proteins and peptides from PeptideAtlas that may be associated, and display relevant literature information from MEDLINE. These associations can be visually explored, and the results directly exported to the experiment design pipeline ATAQS [47] or explored at subsequent levels of visualization with additional data.

**Figure 5:**
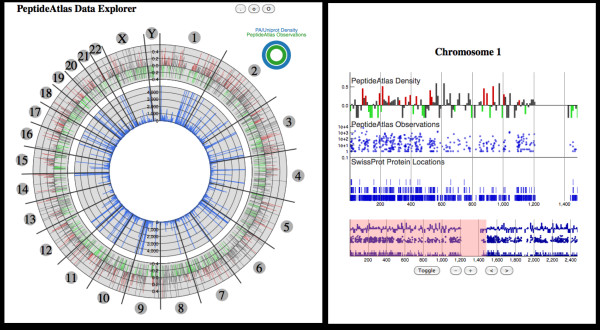
**Visualizing PeptideAtlas**. The PeptideAtlas can be explored through literature and disease associations (Figure 4) as well as through gene centered views. The genome visualization on the left allows a user to mine observed spectra based on chromosome location, and drill down can be undertaken by selecting a location of interest and viewing available genomic and proteomic annotations. The user starts exploration of the repository through the main genome browser to find genes of interest, information about relationships between genes can be displayed in the center of the viewer. Information about the protein products of the genes, relating to information stored in PeptideAtlas, is shown in the concentric rings. The display on the right provides further details about the protein products, including detectability.

**Figure 6:**
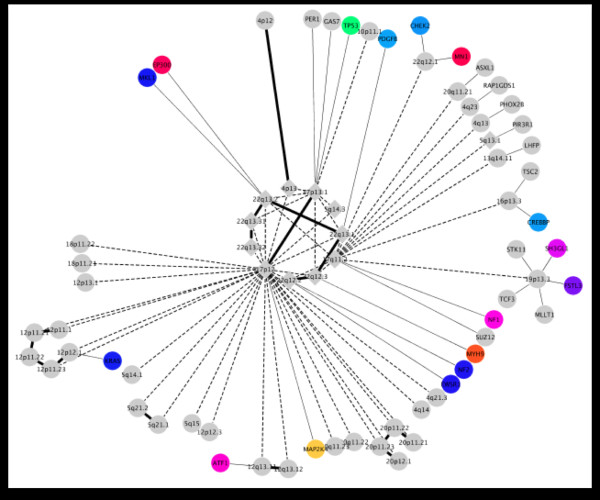
**Cytoscape detectable proteins**. Using Cytoscape, PeptideAtlas information can be overlaid on TCGA data in a network context, allowing users to locate potential biomarkers within the context of a given cancer network. In this instance genes that have been identified as having being important in cancer progression, through changes in gene dosage, are shown. The diamonds show loci of the genes, and the circles show the genes themselves. Information from PeptideAtlas, relating to if it is known if the corresponding protein is detectable, is overlaid.

The next visualization, called CircAtlas (see Figure 5), provides a high level view of observed proteomic data in PeptideAtlas overlaid with genomic information and the concordance between multiple feature types. Again using the circular view of the genome (as in the Figure 2), observed peptides and protein information is overlaid on chromosomal locations. The zoomed in "track" view of a specific chromosome provides more detailed information regarding the observed data and feature annotations. Finally, this data can be integrated with pathway information and TCGA analytical data to provide a Cytoscape [48] network view (see Figure 6) that provides clinically relevant information about possible disease biomarkers.

## Results

One of the important aspects of this work is that the tools must support active research, where the data sets are continually growing and often changing in scale and complexity [49]. This means that the requirements continually change, and this must be factored into the design of visual analytic tools and the associated software technology choice [50,51]. In most cases information visualizations require a costly investment in terms of expertise, user feedback, and developer time. Such investment is beyond most research groups, who must put tools in place quickly, and so often a simple, minimal functionality approach needs to be adopted. Instead of focusing on the best solution, we have found that visualizations must be used together, as each visualization has specific strengths in terms of: ability to work with different sizes of data (e.g. responsiveness); portraying generic aspects of information so that they can be used with multiple data sources; ease of use and understanding; and their suitability for specific tasks (e.g. feature identification).

In addition to good and scalable design, we have found that there are three important facets to the development of visualizations for large scale data repositories which allow for suitable functionality to be put in place:

1. Rapid development and deployment of visualizations, which allows for the development of tools to suit specific tasks through the use of software technologies.

2. Nested task specific views, which allows for the adoption of best information visualization practices without dramatically increasing development time.

3. Common understandable metaphors, which allows for acceptance as intuitive understanding minimizes learning time.

### Rapid development and deployment of visualizations

Visualization is a crucial mechanism for discovering meaningful information from research data. The high volumes, complexity and heterogeneity of the proteomics and genomic data repositories means that representations that simply mirror the data are not appropriate. As research is not a static process, but rather an ongoing dynamic investigative endeavour, the visualizations must be able to deal with highly diverse and continually changing types of information. This means that it is difficult, or in many cases impossible, to provide one visualization that suits all users and usage. Instead, it is more effective to adopt a task based approach, where visualizations are provided for specific tasks (or analysis). If required, common visualizations can then be developed and refined. As data production and research are a constantly moving target, any tools provided to mine the data must be developed as quickly as the data is being produced.

As creating entirely new tools is a time and developer intensive process, using rapid application development (RAD) techniques, appropriate visual analytics can be quickly provided. By adapting tools and using appropriate frameworks, rather than creating entirely new ones, it becomes practical to create views that are specific to a particular task or research direction. For the work discussed in this paper we adopted a number of standard software frameworks. A number of web-based frameworks for both data and visual development were used that enable both rapid application development and rapid data delivery. These include: the Google Data Source libraries for simple, standard data access using SQL database-like language; ExtJS and GXTJS (JavaScript frameworks) for quickly creating intuitive, web-based user interfaces; and web-based visual libraries such as Protovis [52]. Well-known and understood desktop tools such as Cytoscape are also adopted and integrated where appropriate. The use of existing libraries and visual tools has enabled the development of interactive, usable tools (see Figures 4, 5, and 6) within days or weeks rather than months or more.

### Nested task specific views

While it is necessary to enable general exploration, researchers often need to explore the data from a particular starting point. Our visualizations are generally dedicated to a limited set of tasks however, so providing multiple levels of linked visualization for the data becomes necessary (e.g. cross-population, whole genome, chromosomal location). Information visualization has advocated common workflows and scopes for the development of visualizations which are useful when accessing massive data sets. The macro and micro view [53] ideas have more recently evolved into the information seeking mantra [54] workflow (overview, filter, data on demand). Such a workflow offers a practical approach to the delivery of visualizations, so that they can be accessed using desktop and web based tools. However, development of initial "overview" or macro visualizations is not straightforward with large research data sets, and depends largely on the task that is going to be undertaken. For this reason we have typically provide a number of macro views which can be used to start filtering or analyzing the data.

In the PeptideAtlas project the visual tools provide three different "overview" starting points to explore the underlying data. Using the mspecLINE tool (see Figure 4) users can mine literature associations through a disease of interest (e.g. breast neoplasm) and find observed peptide spectra that are linked within the literature. These spectra can then be viewed in the context of the genome or directly viewed. Using the PeptideAtlas Circos visualization, spectra can also be searched by a gene of interest or chromosomal location (see Figure 5) then viewed or exported to other tools. Alternatively, data from other experiments (e.g RNASeq) or network inference analysis can be imported into Cytoscape, and then information about the suitability of the associated proteins to act as biomarkers can be overlaid (see Figure 6).

Using data from TCGA analyses a separate set of macro visualizations provides users with several methods to search through the data. Starting from the Cancer Comparator (see Figure 1), a user can explore gene disruption rates across multiple diseases. This list of genes can then be used to mine a single cancer across multiple samples as within the genome visualization (see Figure 2). The genome visualization can then lead further to specific samples where the gene of interest can be compared across tens of patient samples with the disruptions annotated (see Figure 3).

### Common understandable metaphors

Information visualization provides a means for the non-expert user to mine, interrogate and interact with information at a highly conceptual level. This conceptualization is through a mental model (or metaphor) of the data which is shared by both the developer of the visualization and the end-user. The majority of information visualizations are based around the idea of providing a metaphor which is easily and immediately understandable enables rich interactions with complex and diverse data sets.

In the development of the visualizations discussed above it was found that immediate understanding, typically through the use of common metaphors or positive knowledge transfer, were an important facet of the success of the visualization in portraying information. Rapid understanding and user acceptance of a visualization is important, as it allows scientists to immediately understand what is being portrayed. For example, the Circos plot suffers due to problems associated with the use of atypical non-rectangular based interactions, making it more difficult to use standard mouse drag based operations. However, the familiarity of the metaphor means that people are willing to accept these limitations as they understand both the visual encoding of the information, and are familiar with the layout. Conversely other visualizations, such as the structural variation visualization, are less easy to immediately understand and so general acceptance is limited. In our experience, easy to understand visualizations are those that are used widely, as the researchers themselves typically seek to apply the visualization to other data sets. The metaphors that are familiar are frequently those that are popularly and generically used in visualization (e.g. parallel coordinates) or those that are commonly used in a specific domain (e.g. pathway diagrams, circular genome plots).

## Conclusions

Biology is a big data science with the added complexity that there is no clear understanding as to how the data may be used. Supporting the scale of data that is being generated has led to the development of a number of large scale repositories, and the need to provide visualization tools to mine this data. This paper discusses work that has been undertaken to provide such visual mining tools, and also discusses lessons that have been learned providing tools for both local researchers and the wider community.

We have developed a number of bespoke visual tools, but have preferred to adopt more commonly used designs. In this paper we have discussed a number of visualizations, including: an interactive Circos viewer with context sensitive zooming provided through the track viewer, which shows genomic features and their interactions; Parallel Coordinates, which is used to show and analyze comparisons of genes that are disruptions in different cancers; a table based view, for exploring proteins which are associated with specific diseases and are detectable; and a gene rearrangement viewer, which shows the complexity of localized rearrangements that have been identified through anomalies in read-pairs. We have found that delivery using web technologies is preferable, both due to the low admin requirements and the diverse community using the data. These visualizations each have advantages and disadvantages. The macro views, such as Circos and the parallel coordinates, are relatively easy to understand and use as they provide a high level overview. The nested views, such as the track and gene rearrangement views tend to be more specialized and therefore require some level of learning. Interactivity suffers with the high level views due to the number of items being displayed, this is especially true due to the limitations in web based delivery. Where possible principles of information visualization have been adopted which have been used extensively elsewhere to visualize biological data (e.g. context sensitive displays, multiple encoding of information using intrinsic and extrinsic properties, boundaries and brushing [18,29,55,56]). However, practicalities due to the demands of research (i.e. short time scales, small development teams) means that good design always has to be weighed against rapidly delivering multiple visualizations with the required functionality.

Visual exploration of the large-scale atlas data sets being produced by the PeptideAtlas projects and to support TCGA analysis allows researchers to mine data to find new meaning and make sense at scales from single samples to entire populations. Providing task specific views that allow a user to start from points of interest (from diseases to single genes) allows targeted exploration of thousands of spectra and genomes. As the composition of the atlases changes, and our understanding of the biology increase, new tasks will continually arise. It is therefore important to provide the means to make the data available in a suitable manner in as small a time as possible. We have done this through the use of common visualization workflows, into which we rapidly deploy visual tools which follow common metaphors where possible to assist in understanding. Rapid development of tools and task specific views allows researchers to mine large-scale data almost as quickly as it is produced. Ultimately these visual tools enable new inferences, new analyses and further refinement of atlas level data.

## Authors' contributions

JB and SK conceived of the projects and wrote the manuscript. RK and RB developed the software for visual display. All authors read and approved the final manuscript.
